# Enhanced Immune Response in Immunodeficient Mice Improves Peripheral Nerve Regeneration Following Axotomy

**DOI:** 10.3389/fncel.2016.00151

**Published:** 2016-06-14

**Authors:** André L. Bombeiro, Júlio C. Santini, Rodolfo Thomé, Elisângela R. L. Ferreira, Sérgio L. O. Nunes, Bárbara M. Moreira, Ivan J. M. Bonet, Cesar R. Sartori, Liana Verinaud, Alexandre L. R. Oliveira

**Affiliations:** Department of Structural and Functional Biology, Institute of Biology, University of CampinasCampinas, Brazil

**Keywords:** sciatic nerve, PNS, neuroimmunology, inflammation, axonal regeneration

## Abstract

Injuries to peripheral nerves cause loss of motor and sensory function, greatly affecting life quality. Successful repair of the lesioned nerve requires efficient cell debris removal, followed by axon regeneration and reinnervation of target organs. Such process is orchestrated by several cellular and molecular events in which glial and immune cells actively participate. It is known that tissue clearance is largely improved by macrophages, which activation is potentiated by cells and molecules of the acquired immune system, such as T helper lymphocytes and antibodies, respectively. In the present work, we evaluated the contribution of lymphocytes in the regenerative process of crushed sciatic nerves of immunocompetent (wild-type, WT) and T and B-deficient (RAG-KO) mice. In Knockout animals, we found increased amount of macrophages under basal conditions and during the initial phase of the regenerative process, that was evaluated at 2, 4, and 8 weeks after lesion (wal). That parallels with faster axonal regeneration evidenced by the quantification of neurofilament and a growth associated protein immunolabeling. The motor function, evaluated by the sciatic function index, was fully recovered in both mouse strains within 4 wal, either in a progressive fashion, as observed for RAG-KO mice, or presenting a subtle regression, as seen in WT mice between 2 and 3 wal. Interestingly, boosting the immune response by early adoptive transference of activated WT lymphocytes at 3 days after lesion improved motor recovery in WT and RAG-KO mice, which was not ameliorated when cells were transferred at 2 wal. When monitoring lymphocytes by *in vivo* imaging, in both mouse strains, cells migrated to the lesion site shortly after transference, remaining in the injured limb up to its complete motor recovery. Moreover, a first peak of hyperalgesia, determined by von-Frey test, was coincident with increased lymphocyte infiltration in the damaged paw. Overall, the present results suggest that a wave of immune cell infiltration takes place during subacute phase of axonal regeneration, resulting in transient set back of motor recovery following peripheral axonal injury. Moreover, modulation of the immune response can be an efficient approach to speed up nerve regeneration.

## Introduction

Peripheral nervous system (PNS) demyelinating diseases (Juarez and Palau, 2012; Ripellino et al., 2014; Winer, 2014) and trauma result in action potential impairments with loss of function (Siddique and Thakor, 2014). Successful repair of the lesioned nerve requires efficient cell debris removal, followed by axon regeneration and reinnervation of target organs, a process that is orchestrated by several cellular and molecular events in which glial and immune cells actively participate. After PNS injuries, damaged tissue undergoes clearance and remodeling, the so called Wallerian degeneration. In this process, axons from the distal stump degenerate and the myelin sheaths breakdown, the blood nerve barrier permeability increases and macrophage influx occurs (Vargas and Barres, 2007). Myelin fragments are removed and Schwann cells proliferate and migrate to the lesion site providing structural and physiological support to axon guided growth (Vargas and Barres, 2007; Rotshenker, 2011).

Both the innate and the acquired immune responses are activated after PNS trauma. In the first few days post-injury, Schwann cells remove myelin debris and release neurotrophic factors, cytokines, and chemokines that recruit phagocytes to the lesion site. Tissue clearance is improved by macrophages, which phagocytic capacity is potentiated by pro-inflammatory cytokines released by infiltrating CD4 T cells and opsonins, such as C3b proteins of the complement system (Bruck and Friede, 1990; Sarma and Ward, 2011) and antibodies produced by B lymphocytes. Such molecules are important for rapid myelin removal after nerve injury (Vargas et al., 2010). Tissue clearance is an important step for the regenerative process since axon growth inhibitory proteins, such as the myelin-associated glycoprotein (MAG), are present in the fragmented myelin (Fruttiger et al., 1995; Jessen and Mirsky, 2008). When cell fragments and myelin debris are cleared, the inflammatory response is suppressed by M2 macrophages and T helper (Th) 2 lymphocytes, creating a microenvironment prone to the neuroregenerative process (Gaudet et al., 2011). Although cytotoxic CD8 T cells are found in demyelinating and neurodegenerative diseases, such as multiple sclerosis and amyotrophic lateral sclerosis, their function in disease onset and progression is not fully understood. While some studies suggest a regulatory role of CD8 T lymphocytes on CD4 T cell activity (Zozulya and Wiendl, 2008) and dendritic cell activation (Kashi et al., 2014) during experimental autoimmune encephalomyelitis (EAE), other demonstrate that CD8 T cells aggravate CD4 T cell mediated EAE (Mars et al., 2007). Regarding the PNS regeneration after traumas, very few are known about the role of CD8 T lymphocytes during this process.

In view of the importance of the immune response for nerve regeneration, we evaluated regeneration and motor recovery after crushing the sciatic nerve of mice with distinct levels of acquired immune competency. In this way, we compared the regenerative performance of immunocompetent mice with those lacking mature T and B lymphocytes. Moreover, we analyzed motor function recovery and nociceptive threshold after increasing the immune response by adoptive transference of activated lymphocytes. In this regard, migration to the lesion site was monitored *in vivo* via live imaging analysis.

## Materials and Methods

### Animals

For this study, we used C57BL/6 immunocompetent (wild type, WT) and C57BL/6 recombination activating gene 1 (RAG1)-knockout (KO) mice. During T-and B-cell development, antigen receptor genes undergo somatic recombination to generate a repertoire of cells with distinct antigen receptors, each cell being capable of recognizing one specific antigen. This process depends on the activity of proteins encoded by the RAG1 and the RAG2 genes. In the absence of any of those genes, immune cells do not complete their maturation process, dying by apoptosis before reaching secondary lymphoid organs in the periphery. In this sense, RAG1-KO mice do not produce mature B and T lymphocytes (Mombaerts et al., 1992). Six to eight week old male mice were bred in-house, at the Laboratory of Neural Regeneration, Institute of Biology, State University of Campinas. Mice were kept in appropriate micro-isolators, under a light-dark cycle of 12 h, with controlled temperature and humidity, receiving water and food *ad libitum*. All experiments concerning animal handling were approved by the Institutional Committee for Ethics in Animal Experimentation (Committee for Ethics in Animal Use – Institute of Biology – CEUA/IB/UNICAMP, protocol numbers 2524-1 and 3753-1) and were performed in accordance to the guidelines of the Brazilian College for Animal Experimentation.

### Experimental Procedures

Experimental mice (*n* = 18 each strain) were anesthetized with ketamine (Fort Dodge, USA, 100 mg/kg) and xylazine (König, Argentina, 20 mg/kg) and the left sciatic nerve was crushed at the sciatic notch level, with a flat tip forceps (number 4), being applied a constant pressure during 30 s. Animals were submitted to a functional gait analysis, as described below and at 2, 4, and 8 weeks after lesion (wal), mice (*n* = 6 per time point, each strain) were deeply anesthetized (ketamine 200 mg/kg and xilasin 40 mg/kg) and euthanized by transcardial perfusion with ice cold phosphate buffer saline (PBS) 0.1 M, pH 7.4, followed by cold fixative solution (formaldehyde 10% in PB 0.1 M, pH 7.4). Crushed nerves were dissected out for immunohistochemistry procedures. As negative control, left sciatic nerve of mice that were not submitted to surgery (*n* = 6) were obtained as described above.

### Tissue Preparation and Sampling for Histological Analysis

The two third distal portion of the sciatic nerve (distally to the crushing site) was post-fixed overnight at 4°C, rinsed 3× in PBS 0.1 M and then stored overnight sequentially in 10 and 20% (w/v) sucrose at 4°C. Cryoprotected samples were immersed in tissue freezing medium (Tissue-Tek, Sakura Finetek), frozen in liquid nitrogen cooled *n*-hexane at -35°C and cryostat sectioned (Microm, HM 525). Non-adjacent longitudinal sections (12 μm thick) were mounted in gelatin-coated glass slides, in such a manner that the interval between sections in each slide was 108 μm. In general, 2–3 sections were placed in each slide, depending on the size of the sample. Slides were kept at -20°C up to use (immunohistochemical procedures, H&E or Sudan Black staining).

### Functional Analysis

For the motor function evaluation, we used the automated Cat Walk System (Noldus Inc., Netherlands) as previously described (Barbizan et al., 2013), and calculated the Sciatic Function Index (SFI). In brief, the animal walks on a glass platform that is illuminated in its thickness with green leds, what enhances the footprints, when the paws contact the glass surface. A high-speed camera underneath the walkway records the run and data are transferred to a host computer, where a software analyzes the paw prints. For adaptation, mice were allowed to freely walk in the system during 3 days, 5 min/day, before the real experiments. Control runs were registered before surgery. Data were collected on a daily basis. To calculate the SFI, we employed the following formula (de Medinaceli et al., 1982): SFI = 118.9((ETS – NTS)/NTS) – 51.2((EPL – NPL)/NPL) -7.5, where N, normal or non-operated side; E, experimental or crushed side; PL, print length and TS, total toe spread.

### Mechanical Hyperalgesia

Mice were individually placed in wire grid floor-acrylic cages (10 × 10 × 20 cm high) with a tilted mirror below them, to provide a view of the hind paws. A gradual increasing pressure was applied in the central plantar area of the injured hind paw with a 0.5-mm^2^ polypropylene tip coupled in a handheld force transducer (electronic anesthesiometer, EFF 301 by Insight, Ribeirão Preto, Brazil), evoking flexion reflex and thus the paw withdraw. Pressure intensity was automatically recorded three times for each animal, with an interval of approximately 10 min between each measurement. The baseline paw-withdrawal threshold was obtained before surgery, being 8 g the maximum pressure limit stablished. Mice were evaluated up to 35 days after surgery, as indicated in the results. Data are expressed as the mean of the pressure intensity minus the mean of the baseline pressure values.

### Immunofluorescence and Protein Quantification

Sections were washed with PB 0.1 M and blocked with bovine serum albumin (BSA) 3% (w/v, diluted in PB 0.1 M) for 1 h, at room temperature. Then, samples were incubated overnight at 4°C, with one of the following primary antibodies: mouse anti-neurofilament protein (1:4000, Dako Cytomation, Cat. code: M0762), rabbit anti-growth associated protein 43 (GAP-43, 1:250, Santa Cruz, Cat. code: SC-10786) and rabbit anti-Iba-1 (1:700, Wako, Cat. code: 019-19741). After rinsing, sections were incubated for 45 min, at room temperature, with one of the secondary antibodies: Cy3-AffiniPure donkey anti-mouse IgG (1:500, Jackson ImmuneResearch, Cat. code: 715-165-150) and Cy3-AffiniPure donkey anti-rabbit IgG (1:500, Jackson ImmuneResearch, Cat. code: 715-165-152). Sections were rinsed in PBS and coverslips were mounted with anti-fading media (ProLong^®^ Gold anti-fading reagent, Invitrogen, Cat. code: P36930). All antibodies were diluted in PB 0.1 M solution containing BSA 1% (w/v) and Triton X-100 0.2% (v/v). For detection of autoantibodies produced by WT mice used for the adoptive cell transfer (bellow), nerve sections from unlesioned RAG-KO mice were incubated with serum (1:30) obtained from unlesioned or sciatic nerve crushed WT mice (14 dal) overnight at 4°C, followed by CY3 anti-mouse IgG, as previously described. As internal control, sections were submitted to the same procedures, being incubated with PBS instead of serum. For protein quantification, sections were analyzed in a fluorescence microscopy (Nikon Eclipse TS100 microscope connected to a Nikon DXM1200F digital camera or Leica DM5500B microscope coupled with a Leica DFC345 FX camera). For each sample, distal to the lesion site, five equidistant representative pictures were obtained. The integrated density of pixels (IDP) was obtained with the ImageJ software (version 1.45s, National Institute of Health, USA), applying the enhance contrast and density slicing feature in a fixed area of each picture (490,000 pixels^2^). The IDP mean values from each sample were considered as the raw data. Results are expressed as the ratio of the mean values of the operated groups by their respective controls (non-operated) when no difference in the IDP between the unlesioned groups was observed. If differences between the control groups were observed, data are shown as the raw IDP mean values.

### Lymphocyte Adoptive Transfer

**FIGURE 1:**
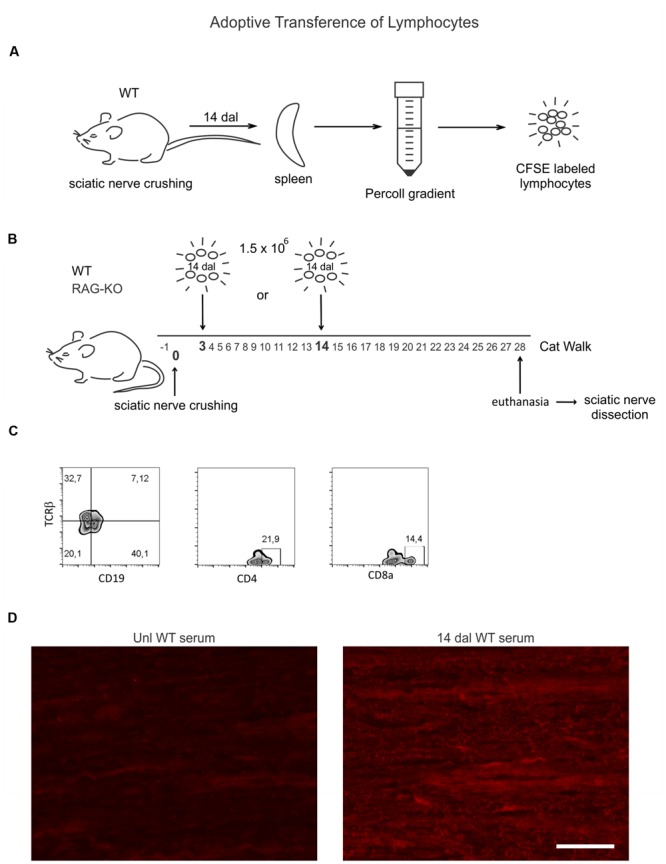
**Wild-type (WT) lymphocyte adoptive transfer procedures and cell phenotyping. (A)** Spleen lymphocytes of WT mice at 14 days after sciatic nerve crushing (*n* = 4) were isolated by Percoll gradient and labeled with a fluorescent probes (CFSE). **(B)** 1.5 × 10^6^ cells were injected via retro-orbital plexus in WT and RAG-knockout (KO) mouse groups at 3 (*n* = 5) or 14 (*n* = 5) days after nerve crushing. Recipient mice were submitted to the CatWalk test before (-1 dal) and after (3–28 dal) surgery, being euthanized at 28 dal and the sciatic nerve dissected out for immunostaining evaluation. **(C)** CFSE unlabeled isolated lymphocytes were phenotyped by flow cytometry, as indicated. **(D)** Unlesioned RAG-KO mouse nerve sections were incubated with serum from unlesioned or sciatic nerve crushed WT mice (14 dal), indicating the production of antibodies against components of the peripheral nerve by injured WT mice (right panel). Scale bar: 50 μm.

Unilateral sciatic nerve crushed WT mice (*n* = 4) were euthanized by halothane inhalation at 14 days after lesion (dal). Spleens were collected and dissociated in cold PBS with a 70 μm cell strainer. Lymphocytes were isolated by Percoll (Sigma–Aldrich, Cat. code: P1644) gradient according to the manufacturer instructions, labeled with carboxyfluorescein succinimidyl ester (CFSE, 1.25 μM, Sigma–Aldrich, USA) and intravenously injected by the retro-orbital route (1.5 × 10^6^) in sciatic nerve-lesioned mice, under deep anesthesia (ketamine 25 mg/kg and xylazine 5 mg/kg). WT and RAG-KO mice were divided into two groups: one that received cells at 3 dal (*n* = 5) and another that received cells at 14 dal (*n* = 5). One day before crushing and from 3 to 28 dal, recipient mice were submitted to the CatWalk functional analysis evaluation, as described above. At 28 dal, all animals were euthanized and the damaged nerves were collected for immunohistochemistry evaluation, as previously described. A scheme summarizing this experiment is shown in **Figure 1**.

### Flow Cytometry

After isolation by Percoll gradient for adoptive transfer (see above), sets of unlabeled lymphocytes were incubated (30 min, 4°C) with antibodies, as follows: (i) anti-mouse CD8a FITC (0.5 μg, eBioscience, Cat. code: 11-0081); (ii) anti-CD4 PerCP-Cy5.5 (0.5 μg, eBioscience, Cat. code: 45-0042-82); (iii) anti-TCRβ PE-Cy7 (0.5 μg, eBioscience, Cat. code: 25-5961-82), and (iv) anti-CD19 FITC (0.5μg, BD, Cat. code: 553785). Cells were washed and 50,000 events were acquired from each sample in a cytometer (Gallios, Beckman Coulter) and analyzed with FlowJo 10.0.5 software (Tree Star Inc., Ashland, OR, USA).

### *In Vivo* Imaging

Percoll isolated lymphocytes obtained as for the adoptive cell transfer (see above) were labeled with nanocrystals according to the manufactory’s instructions (Qtracker 800 Cell Labeling Kit, Molecular Probes, Cat. code: Q25071MP). Sciatic nerve-crushed WT (*n* = 3) and RAG-KO (*n* = 5) mice were injected with labeled cells (1.5 × 10^6^) via the retro-orbital route at 3 days after surgery. Groups of WT (*n* = 5) and RAG-KO mice (*n* = 5) that were submitted to all the surgical procedures without nerve crushing (Sham control) also received labeled lymphocytes. Cell migration to the site of injury was accompanied in different time points, as indicated in the results, using an *in vivo* imaging analyzer (*in vivo* FXBRO, Bruker, TX, USA). For that, fluorophore excitation and emission wavelengths were 710 and 790 nm, respectively, and the imaging time was 1.5 min. Additionally, mice were X-rayed for the anatomical localization of the labeling. During the imaging procedures, mice were kept under anesthesia (isoflurane 3%). To exclude possible unspecific labeling, a negative control mouse (operated, but without labeled lymphocytes) was imaged together with sham and cell-recipient mice. Special care was taken regarding the fur, that was completely removed from the interest area, as well as the skin, that was cleaned to avoid fluorescence interfering materials (food and cage substrate).

### Statistical Analysis

Data were analyzed by the one-way analysis of variance (ANOVA) when comparing groups belonging to the same mouse strain through the experiment time course, or by the two-way ANOVA when comparing WT and RAG-KO groups among them along the time course of the experiment. In both ANOVA approaches, Bonferroni’s multiple comparison tests were carried out as *post hoc* test. Data are expressed as mean ± standard error mean (SEM) and *P* values < 0.05 were considered significant. All statistical analyses were performed using GraphPad Prism (version 4.00, GraphPad Software, San Diego, CA, USA).

## Results

### Axonal Regeneration

**FIGURE 2:**
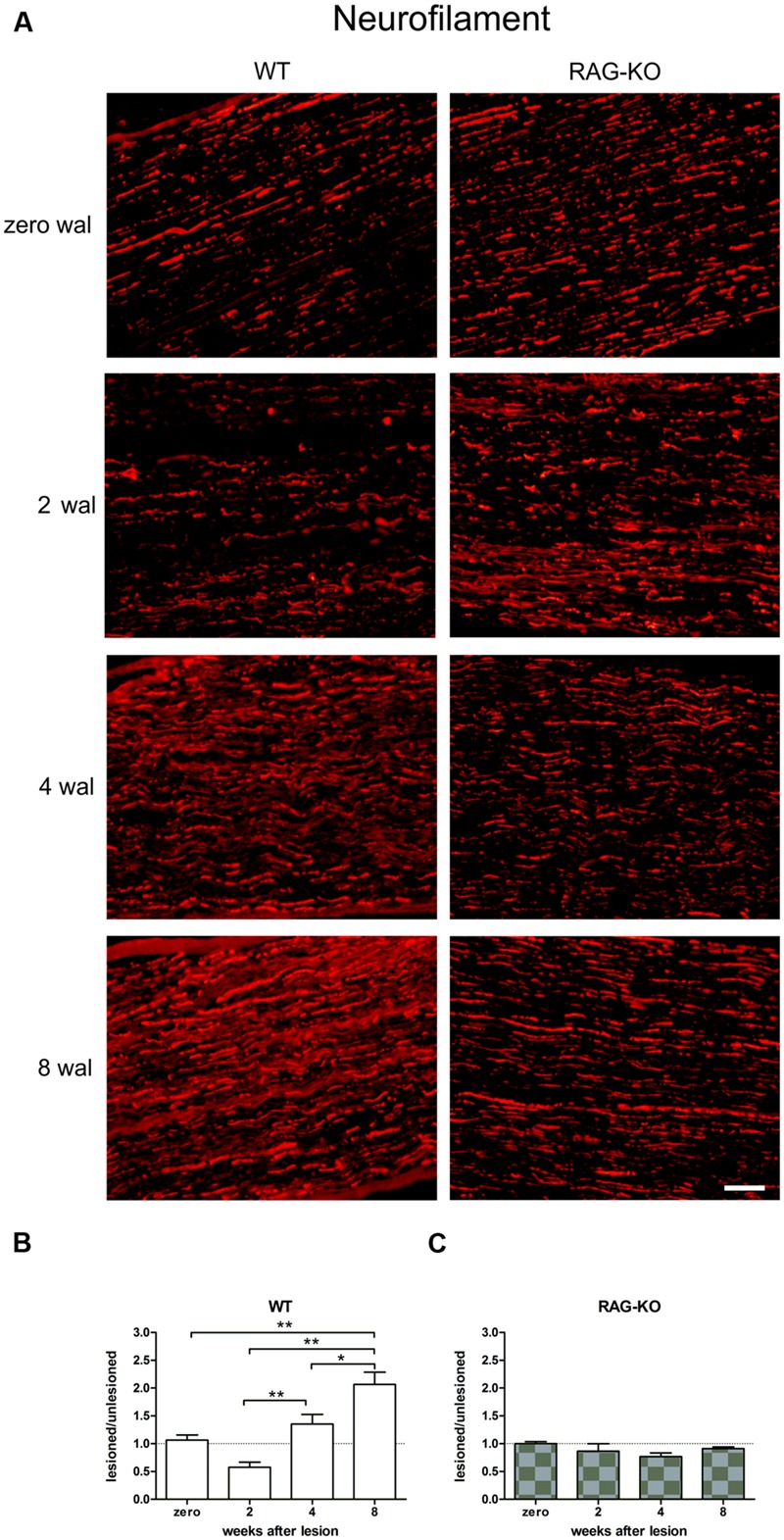
**Crushed sciatic nerve axonal regeneration.** Crushed sciatic nerves from WT and RAG-KO mice were dissected out 2, 4, and 8 weeks after surgery and immunolabeled against neurofilaments (axonal cytoskeleton protein), which expression was quantified by the integrated density of pixels method. Unlesioned mice were used as control. **(A)** Representative images of the neurofilament labeling in mouse strains throughout the time course of the experiments. Zero represents unlesiond mice. wal, weeks after lesion. Scale bar: 50 μm. Ratio of the integrated density of pixels of the nerve crushed groups by each respective unlesioned control group for WT **(B)** and RAG-KO mice **(C)**. Data are presented as mean ± SEM. *n* = 6 in each time point for both strains. ^∗^*p* < 0.05; ^∗∗^*p* < 0.01 according to the one-way ANOVA, followed by Bonferroni post-test.

The immunolabeling evaluation revealed an upregulation of neurofilament expression during the regenerative process following sciatic nerve crushing in the WT samples, reaching the peak at 8 wal (**Figures 2A,B**; Supplementary Table S1). However, no change in the RAG-KO tissue was observed in the analyzed time points (**Figures 2A,C**; Supplementary Table S1). Comparison between mouse strains revealed that WT mice presented more neurofilaments than RAG-KO at 4 wal (*p* < 0.01; **Figures 2B,C**) and 8 wal (*p* < 0.001; **Figures 2B,C**). Of note, no difference in the labeling was seen between the mouse lineages under basal conditions.

**FIGURE 3:**
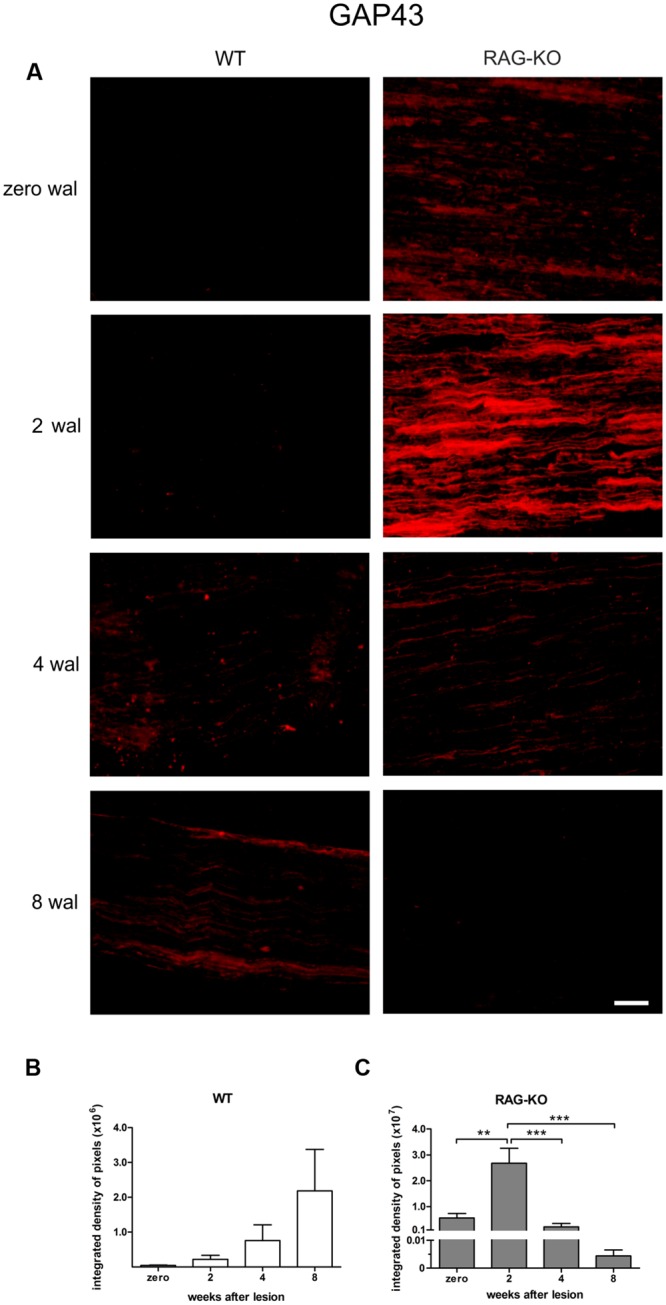
**Expression of growth-associated protein (GAP) 43 during the nerve regenerative process. (A)** Representative images of GAP-43 labeling in WT and RAG-KO sciatic nerves at 2, 4 and 8 weeks after crushing. Zero represents nerve sections from non-operated mice. wal, weeks after lesion. Scale bar: 50 μm. Integrated density of pixels (arbitrary units) of GAP-43 immunolabeling in the sciatic nerve of WT **(B)** and RAG-KO mice **(C)**. Note the higher immunolabeling in the RAG-KO group under basal conditions (0 wal), compared to the WT group. Data are presented as mean ± SEM. *n* = 6 in each time point for both strains. ^∗∗^*p* < 0.01; ^∗∗∗^*p* < 0.001 according to the one-way ANOVA, followed by Bonferroni post-tests.

Although the neurofilament baseline level recovery in RAG-KO nerves at 2 wal was suggestive of a faster regenerative process, it could also indicate a slower removal of axon debris. In order to address this question, we analyzed the expression of the growth associated protein 43 (GAP-43), which is expressed by regenerating axons, especially in growth cone membranes (Skene et al., 1986). In the WT, GAP-43 levels increased throughout the time course (*p* < 0.05, **Figures 3A,B**, Supplementary Table S2), while it peaked in RAG-KO at 2 wal, decreasing thereafter (**Figures 3A,C**; Supplementary Table S2). Differences in the integrated density of pixels between the strains were observed at 2 wal, when RAG-KO presented more GAP-43 than WT (*p* < 0.001; **Figures 3B,C**). Under basal conditions, GAP-43 expression was higher in RAG-KO than in WT mice (*p* < 0.01; **Figures 3B,C**). These data are suggestive of a faster axonal recovery in the immunodeficient mice.

**FIGURE 4:**
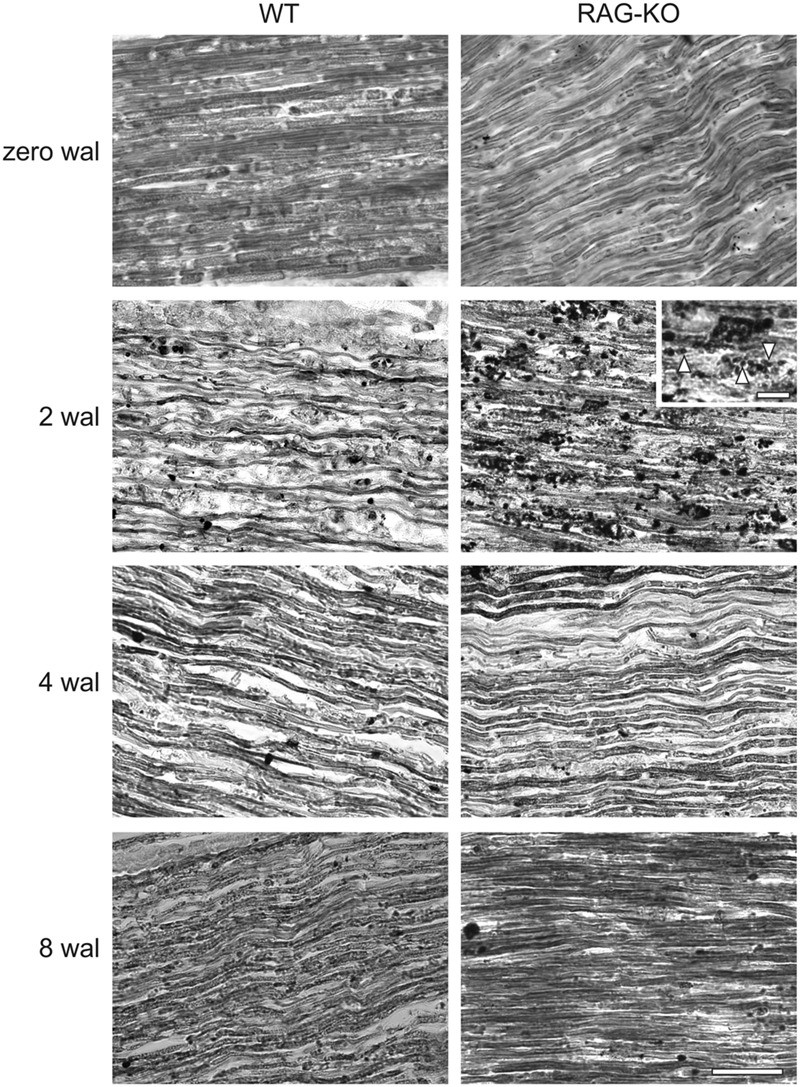
**Myelination after crushing of the sciatic nerve.** Sudan black staining of uninjured and crushed sciatic nerves of WT and RAG-KO mice. Note the high amount of lipid fragments in the RAG-KO nerve at 2 wal (inset, arrowheads). Zero represents nerves from non-operated mice. wal, weeks after lesion. Scale bar: 50 μm; inset: 10 μm.

Myelin staining revealed low number of myelinated axons at 2 wal for both mouse strains (**Figure 4**), being this pattern reversed at 4 wal, when more myelinated axons were seen (**Figure 4**). Interestingly, at 2 wal more myelin fragments or cell debris were observed in the RAG-KO nerve as compared to the WT (**Figure 4**).

### Peripheral Cell Infiltration in the Crushed Sciatic Nerve

**FIGURE 5:**
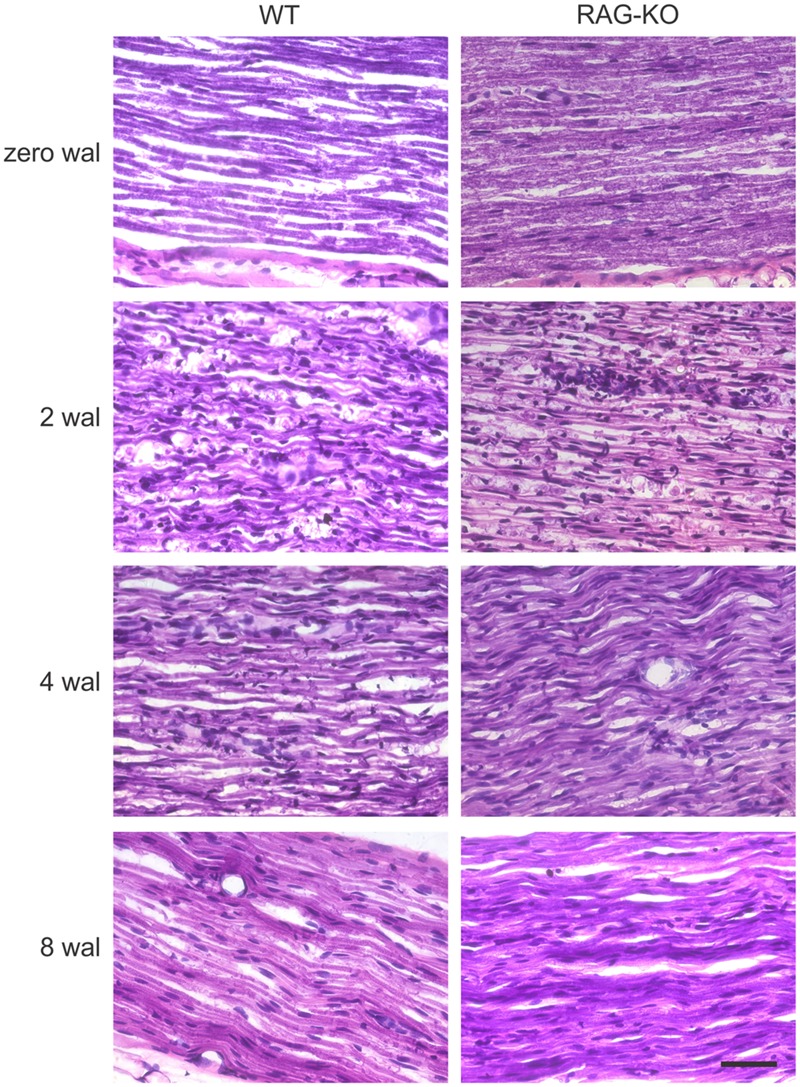
**Peripheral cell infiltration after crushing of the sciatic nerve.** H&E staining of uninjured and crushed sciatic nerves of WT and RAG-KO mice. Note the high amount of infiltrating cell nuclei at 2 wal, which progressively decreased up to 8 wal. Zero represents nerves from non-operated mice. wal, weeks after lesion. Scale bar: 50 μm.

**FIGURE 6:**
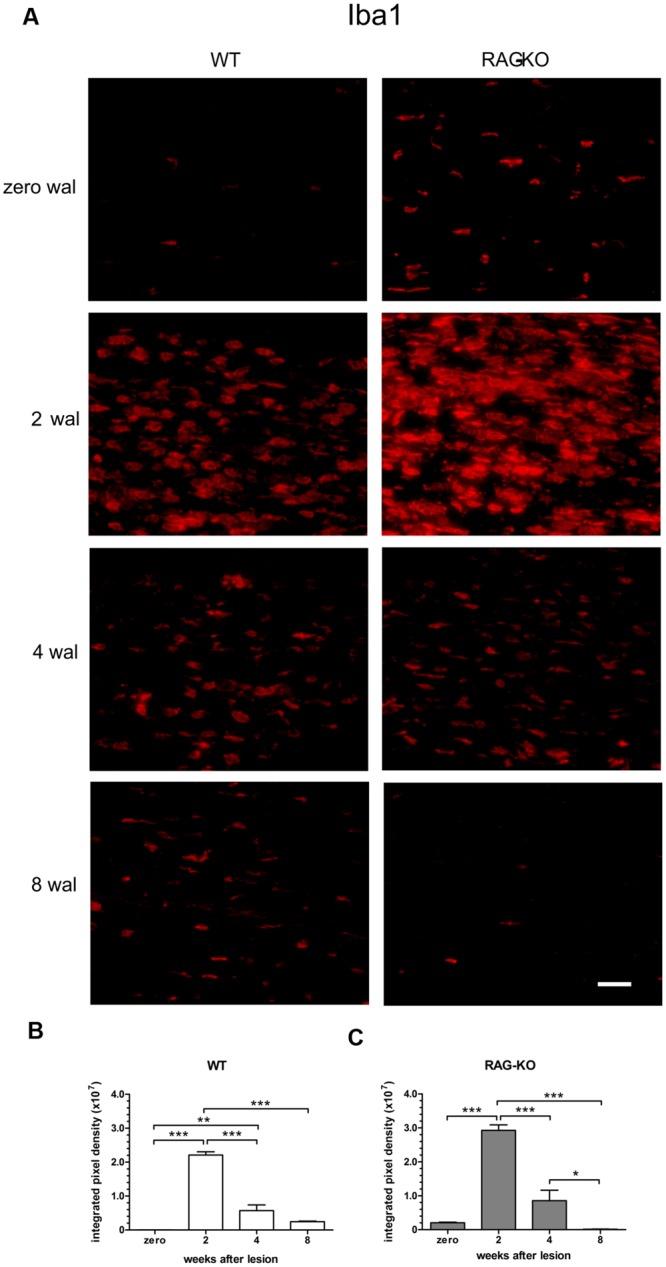
**Macrophage infiltration in the sciatic nerve after crushing.** Intact or crushed sciatic nerves of WT and RAG-KO mice were imunolabeled against the macrophage marker Iba1 and submitted to the quantification method of the integrated density of pixels. **(A)** Representative images of Iba-1 labeled macrophages from both mouse strains through the time course of the study. Zero represents unlesiond mice. wal, weeks after lesion. Scale bar: 50 μm. Integrated density of pixels (arbitrary units) of Iba-1 immunolabeling in the sciatic nerve of WT **(B)** and RAG-KO mice **(C)**. Note that under basal conditions, KO mice have more macrophages than WT. Data are presented as mean ± SEM. *n* = 6 in each time point for both strains. ^∗^*p* < 0.05; ^∗∗^*p* < 0.01; ^∗∗∗^*p* < 0.001 according to the one-way ANOVA, followed by Bonferroni post-test.

After injury, the establishment of an inflammatory immune response is an essential step for the recovery of the tissue homeostatic state. As expected, we observed a high amount of infiltrating cells in the WT and RAG-KO nerves at 2 weeks after crushing, decreasing along the time, as regeneration occurred (**Figure 5**). When investigating the presence of macrophages in the injured nerve, we also observed an increased amount of cells at 2 wal in both mouse strains, decreasing thereafter (**Figures 6A–C**; Supplementary Table S3). However, comparison between strains revealed that, at the peak of macrophage infiltration, namely 2 wal, RAG-KO mice presented around 30% more Iba-1 immunolabeled cells than the WT animals (*p* < 0.01; **Figures 6B,C**). Interestingly, under basal conditions we observed 50-fold more Iba-1 immunoreactivity in RAG-KO nerve sections than in the WT tissue (*p* < 0.001; **Figures 6B,C**), suggesting a stronger innate immune response as a compensatory mechanism to the impaired adaptive immunity carried by the KO animals.

### Functional Analysis

**FIGURE 7:**
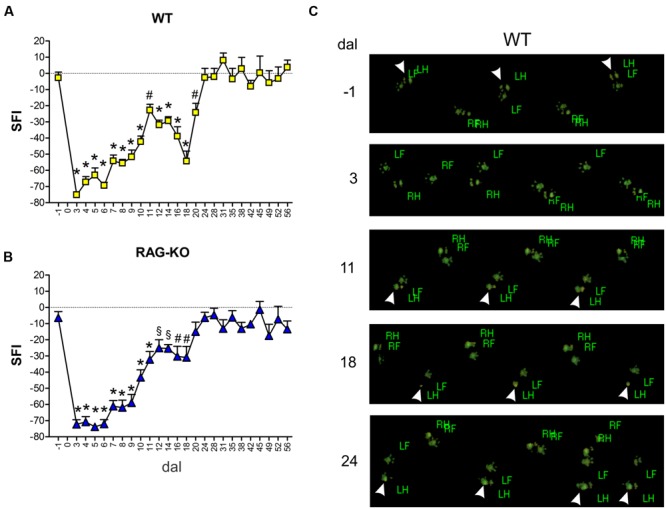
**Motor function evaluation.** WT and RAG-KO mice were submitted to the automated walking track test before (-1 dal) and after (3–56 dal) the left sciatic nerve crushing. Sciatic function index (SFI) values (arbitrary units) of WT **(A)** and RAG-KO mice **(B)** along the time course. Data are presented as mean ± SEM. *n* = 18 (-1 to 14 dal), *n* = 12 (16–28 dal), *n* = 6 (31–56 dal). ^§^*p* < 0.05; ^#^*p* < 0.01; ^∗^*p* < 0.001 in comparison to mice before surgery (-1 dal), according to the one-way ANOVA followed by Bonferroni post-test. **(C)** WT footprints before (-1 dal) and after (3–24 dal) the surgery. Arrowheads indicate left hind limb footprints. dal, days after lesion; RF, right front; RH, right hind; LF, left front; LH, left hind.

Besides the faster axonal regenerative response presented by the KO animals, we were interested in investigating the functional recovery, assessed by an automated walking track test system. The functional analysis demonstrated that WT and RAG-KO reestablished lesioned hind limb movements after 20 and 18 days post-injury, respectively (**Figures 7A,B**). Interesting, while KO animals recovered motor function in a progressive fashion (**Figure 7B**), WT mice relapsed from days 11 to 18 after crushing, successfully recovering thereafter (**Figure 7A**). Of note, 18 dal is the only time point in which WT group differs from RAG-KO mice (*p* < 0.05, according to the 2-way ANOVA). **Figure 7C** shows differences in the left hind limb footprint pattern of WT mice in different time points.

### Adoptive Transference of Lymphocytes Interferes in the Motor Recovery Following Nerve Injury

Relapse of motor recovery in WT group at 18 days after nerve crushing indicates that the adaptive immune response is involved, since mutant mice recovered without subtle regression (**Figure 7**). To address if such finding could be reproduced in RAG-KO mice, we transferred lymphocytes from the spleen of crushed sciatic nerve WT mice to immuno-competent and deficient animals. After injuries, antigens are presented to lymphocytes in the peripheral lymphoid organs, such as the spleen and lymph nodes, where those cells undergo clonal expansion before migrating to the lesion site. In this way, the spleen is a good source of lymphocytes with specificity to the crushed nerve antigens. Since lymphocyte proliferation requires time, cells were obtained at 14 days after sciatic nerve crushing, a period coincident with a substantial influx of such cells into the damaged nerve (Gaudet et al., 2011). A phenotypic analysis of those splenic cells reveled that approximately 40% of them were B lymphocytes (CD19^+^), while 22% were CD4 T cells and 14% were CD8 T cells (**Figure 1C**). In order to evidence the production of antibodies against components of the crushed nerve by the transferred B cells, we incubated RAG-KO intact nerve tissue with serum obtained from the lymphocyte donor WT mice, as well as from non-operated control WT animals. According to **Figure 1D**, the intact nerve was largely immunolabeled when incubated with serum from operated mice. Of note, natural antibodies were also found in the serum of unlesioned mice (**Figure 1D**). No immunolabeling was visualized when the tissue was incubated with PBS instead of serum, followed by CY3 anti-mouse IgGs (not shown).

**FIGURE 8:**
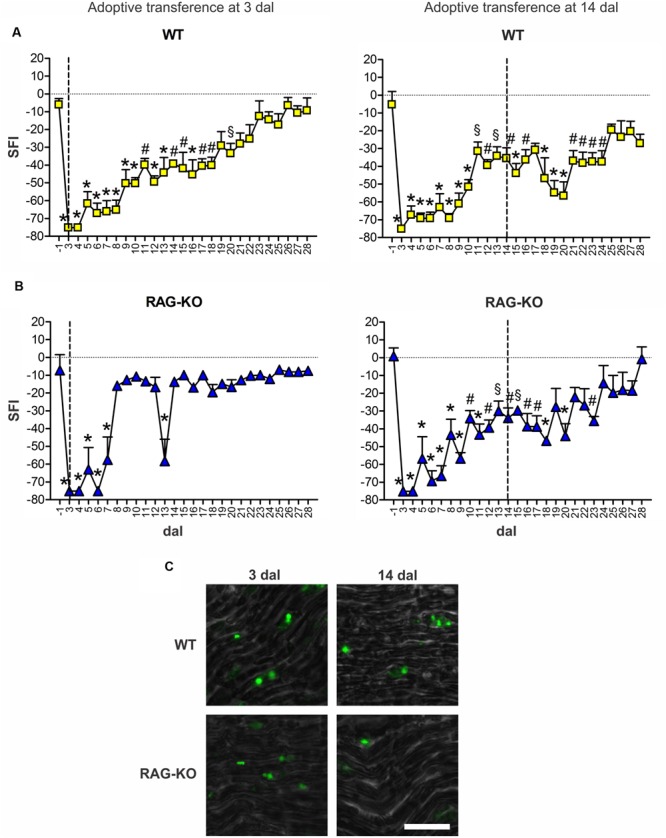
**Motor function interference by adoptive transference of WT lymphocytes.** WT **(A)** and RAG-KO **(B)** mice that adoptively received WT lymphocytes at 3 or 14 days after sciatic nerve crushing were submitted to the automated walking track test before (-1 dal) and after the surgery (3–28 dal). At 28 dal, nerves were dissected out and the CSFE labeled lymphocytes were observed **(C)**. Data are presented as mean ± SEM. *n* = 5 for all cell recipient mouse groups. ^§^ *p* < 0.05; ^#^*p* < 0.01; ^∗^*p* < 0.001 in comparison to mice before surgery (-1 dal), according to the one-way ANOVA followed by Bonferroni post-test. SFI, sciatic function index; dal, days after lesion. Scale bar: 30 μm.

Boosting the immune response by the adoptive transference of lymphocytes to WT animals at 3 dal did not worsen motor recovery, instead, it prevented motor loss relapse (**Figure 8A**). On the other hand, the late adoptive transference of lymphocytes did not cause the same effects. WT mice that received cells at 14 dal presented motor loss from 17 to 20 dal and impaired motor recovery up to 24 dal (**Figure 8A**). Regarding RAG-KO mice, cell transfer at 3 dal improved motor recovery, which SFI baseline values (-1 dal) were quickly reached at 8 dal (**Figure 8C**), while in the non-transferred control group it occurred more than 10 days later, i.e., longer than 18 dal (**Figure 7B**). Interesting, recipient RAG-KO group presented a subtle regression at 13 dal, fully recovering movements from day 14 on (**Figure 8C**). Transference of lymphocytes at 14 dal in RAG-KO mice had an opposite effect, impairing motor recovery up to 23 dal (**Figure 8C**), i.e., 5 days more than the control group (**Figure 7B**). When analyzing the crushed nerve at the end of the experimental period (28 dal), we observed that lymphocytes in fact migrated to the damaged tissue when injected at 3 or 14 dal, in both immune-competent and immune-deficient animals (**Figure 8C**).

### Lymphocyte Migration to the Crushed Nerve and Paw Sensibility Following Adoptive Cell Transference

**FIGURE 9:**
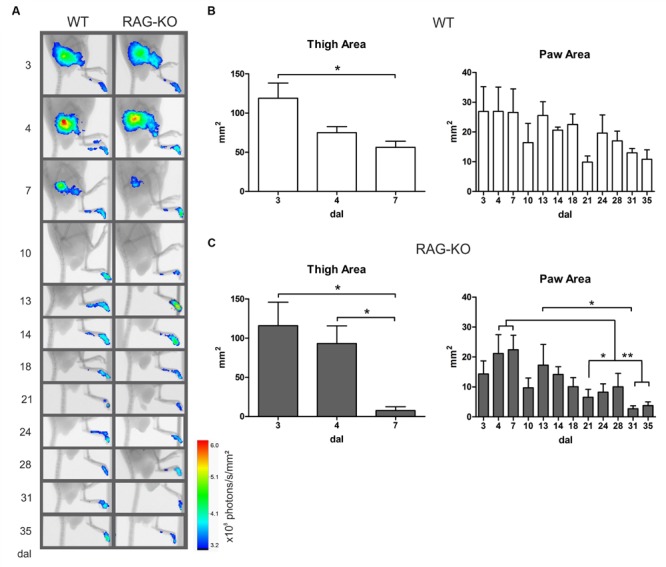
**Lymphocyte migration to the crushed nerve following cell adoptive transfer.** WT and RAG-KO mice were submitted to sciatic nerve crushing and at 3 days after surgery, they received nano crystal-labeled lymphocytes. Cell migration was followed from 3 h after transference up to 35 days after lesion **(A)** and the labeling area in the thigh and paw was quantified in WT **(B)** and RAG-KO **(C)** groups. Data are presented as mean ± SEM. ^∗^*p* < 0.05; ^∗∗^*p* < 0.01, according to the one-way ANOVA followed by Bonferroni post-test. *n* = 3 for WT; *n* = 5 for RAG-KO. dal, days after lesion.

Better results on motor recovering were obtained when cells were transferred in the beginning of the degenerative process (**Figure 8**). To determine when lymphocytes migrated to the damaged tissue and the period they acted there, cells were labeled immediately before transference (at 3 dal) and tracked *in vivo* up to 35 dal, when motor function was fully reestablished. In WT and RAG-KO groups, lymphocytes were seen in the crushed nerve as soon as 3 h after transference, remaining in the tissue until the end of the experiment (**Figure 9A**). Up to 7 dal, cells were present in both the thigh and the paw, after this period, being visualized only in the paw (**Figure 9A**). Thigh labeled area measurements showed us that in the WT group there was a decrease from 119 to 56 mm^2^ from day 3 to 7 after lesion (*p* < 0.05), while in the RAG-KO group, it was reduced from 116 to 8 mm^2^ in the same period (*p* < 0.05). Of note, from day 4 to 7 no significant decrease in the labeling area was observed in the WT thigh, on the contrary of that seen in the RAG-KO group (**Figures 9B,C**; Supplementary Table S4), suggesting the need of lymphocytes for a prolonged period in the regenerative process of the WT nerve. Regarding the paw labeled areas, both the groups presented oscillations through the time course (*p* < 0.01 for WT and *p* < 0.001 for RAG-KO, according to the two-way ANOVA) and were different between then in a global analysis (*p* < 0.001, according to the two-way ANOVA). Both presented a similar kinetics in the labeling area that picked on day 7 after crushing (**Figures 9A–C**; Supplementary Table S5). Curiously, a second and a third wave of infiltrating lymphocytes were observed for both lineages between days 10 and 21, and 21 and 35 after crushing (**Figures 9B,C**; Supplementary Table S5). No difference in the labeling intensity was observed between the lineages for the analyzed parameters (data not shown).

**FIGURE 10:**
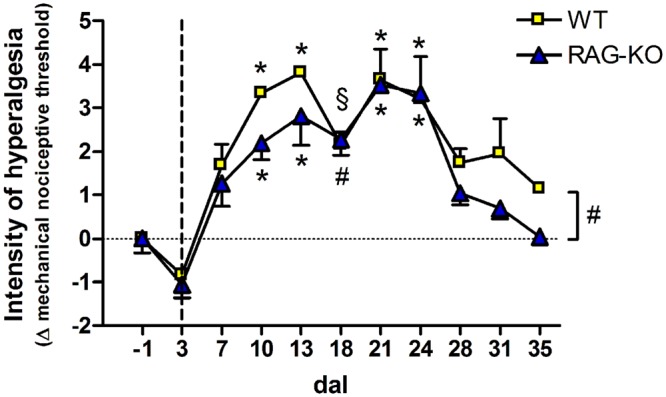
**Hyperalgesia of crushed-nerve mice following adoptive transfer of lymphocytes.** Lymphocyte recipient WT and RAG-KO mice were submitted to the electronic Von Frey test to measure the paw sensibility before (-1 dal) and after sciatic nerve crushing, up to 35 dal. Values below zero indicate analgesia while above zero, hyperalgesia. The vertical dashed line signalizes the adoptive cell transfer day. Data are presented as mean ± SEM. ^§^ *p* < 0.05; ^#^p < 0.01; ^∗^*p* < 0.001 in comparison to mice before surgery (-1 dal), according to the one-way ANOVA followed by Bonferroni post-test. *n* = 3 for WT; *n* = 5 for RAG-KO dal, days after lesion.

When analyzing the paw sensibility, it was possible to observe that animals presented no sensibility at 3 dal (**Figure 10**), a period they were completely paralyzed (**Figure 8**). As expected, both groups recovered sensibility baseline levels throughout the time course (**Figure 10**). Interestingly, a first peak of hyperalgesia was observed at 13 dal for WT and RAG-KO mice (**Figure 10**), coincident with the second peak of infiltrating lymphocytes in the damaged paw (**Figures 9B,C**) and with the subtle regression of movements displayed by RAG-KO mice after cell transference (**Figure 8B**). A second peak of hyperalgesia was observed at 21 days after crushing for both lineages (**Figure 10**), however, when nerve regeneration must be in an advanced stage and lymphocyte infiltration in the paw is reduced (**Figures 9B,C**).

## Discussion

Neuronal and glial responses to PNS damage have been addressed in many studies. Nevertheless, the cellular and molecular mechanisms behind the neuroregenerative process still are not fully understood. More recently, attention has been given to the possible overlap between immune and neural response to injury, so that many molecules have been shown to develop dual roles, participating in the classical immune response, but also in the interaction between neurons and surrounding glia. In this way, it has become more evident that the nervous and immune system interact and overlap mechanisms that in turn interfere in the success or failure of the regeneration process after neuronal injury (Gaudet et al., 2011; Rotshenker, 2011; Shastri et al., 2013; Xanthos and Sandkuhler, 2014).

An increasing number of studies show a neuroprotective role for T cells, in special CD4 T lymphocytes, after damage to the nervous system (Hendrix and Nitsch, 2007; Walsh et al., 2014). Following mouse facial nerve axotomy, Serpe et al. (2003) demonstrated that motorneuron survival is higher in WT mice than in CD4-KO and RAG-2-KO mice. However, neurons are rescued from death to the same level of WT animals when both KO groups were reconstituted with CD4 T lymphocytes. Besides that, the lack of CD8 T cells or B cells did not worsen neuronal survival, which was also not improved in RAG-2-KO mice reconstituted with either CD8 T cells or B lymphocytes (Serpe et al., 2003). Moreover, neuroprotection was mediated by autoreactive CD4 T cells independently of their ability to secret brain derived neurotrophic factor (BDNF) (Xin et al., 2012). In order to evaluate the involvement of the acquired immune-response after damage of the sciatic nerve, we employed T and B cell deficient mice, but contrarily to our expectations, no deficit in the regenerative process was observed in those animals, which presented even more neurofilaments and axon growth proteins in the initial phase of the regenerative process. Accordingly, improved motor recovery and enhanced axon regrowth and myelination were observed in RAG-2-KO animals submitted to either nerve transection followed by tubulization (Mehanna et al., 2014) or to spinal cord compression (Wu et al., 2012), both the cases at six weeks after surgery. In a mouse model of Charcot–Marie–Tooth disease, a demyelinating peripheral neuropathy, the lack of mature T and B lymphocytes was also beneficial, decreasing the percentage of demyelinated axons, supernumerary Schwann cells and periaxonal vacuoles in the PNS (Kobsar et al., 2003).

Additionally, the immunodeficient mice herein studied presented, under basal conditions, significantly more macrophages than the WT animals, as well as in the initial phase of the regenerative process. As largely known, macrophages and Schwann cells are important for myelin removal during Wallerian degeneration, a process that precedes the axonal regeneration when considering the presence of axon growth inhibitory proteins in the myelin debris. In the first few days post-injury, Schwann cells are responsible for myelin clearance of the distal stump and around 3 dal, phagocytosis is improved by monocyte-derived macrophages that enter the damaged nerve, peaking from 14 to 21 days post-injury (Vargas and Barres, 2007). Although Schwann cells may remove myelin in the absence of infiltrating macrophages (Perry et al., 1995), it has been shown that myelin clearance during Wallerian degeneration is delayed in mice with impaired macrophage infiltration due to the lack of chemotactic substances (Liefner et al., 2000; Sugiura et al., 2000). Moreover, myelin phagocytosis by macrophages is strongly dependent on the complement receptor-3 (CR-3) (Bruck and Friede, 1990) and antibodies against myelin compounds (Vargas and Barres, 2007), being the nullity of immunoglobulin in B cell deficient mice also responsible for impaired axon regeneration after sciatic nerve crushing (Vargas et al., 2010). Aside the phagocytic capacity, macrophages also produce neurotrophins, such as insulin-like growth factor (IGF) (Suh et al., 2013), BDNF, neurotrophin 3 (NT-3) (Barouch et al., 2001a) and nerve growth factor (NGF) (Barouch et al., 2001b) and also stimulate production of NGF by Schwann cells via interleukin-1 (Lindholm et al., 1987). More recently, it was reported that under hypoxia conditions, macrophages release the pro-angiogenic factor VEGF-A, stimulating polarized blood vessels formation in the damaged nerve (Cattin et al., 2015). Moreover, Schwann cells use those new vessels as tracks to guide their migration across the wound, taking the growing axons with them (Cattin et al., 2015). In view of the importance of macrophages in the neuronal regenerative process, it is reasonable to attribute part of the improved axonal regeneration displayed herein by the KO mice to the increased amount of macrophages in the acute period post-injury, rather than the lack of lymphocytes.

One interesting finding of the present work is the motor function loss relapse presented by WT, but not by the immune-deficient mice after two weeks following peripheral nerve injury. Although the most remarkable difference between those animals is the impaired adaptive immune response carried by the KO mice, our data also point to an enhanced innate immune response leading to a higher macrophage infiltration in the first two weeks after nerve crushing in RAG-KO animals. Although inflammation is necessary to reestablishing tissue homeostasis, it can be harmful when exacerbated due to the intensity and duration of the antigenic stimuli. Pro-inflammatory mediators and free radicals may cause damage to the nervous tissue, impairing axonal conductivity or even causing retrograde neuronal death (Hendriks et al., 2005; Brown, 2007). In this sense, the motor function loss relapse presented by WT mice seems to be linked to the effects of the adaptive immune response, possibly leading to transitory impairment of axonal conductivity or even pain that hampered functionality of the lesioned limb. When transferring activated lymphocytes to WT mice at 3 days after crushing, we anticipated the acquired immune response that should have potentiated the innate immunity, improving tissue clearance and thus resulting in a milder inflammation thereafter, causing no loss of movements at 18 days after crushing.

The benefits of the early cell transfer became more evident in RAG-KO mice, which recovered movements in a few days after cell injection, accompanied by an immediate lymphocyte migration to the lesion that should have stimulated phagocytic cell activation and improved tissue clearance. Additionally, lymphocytes could also have repopulated the secondary lymphoid organs, migrating to the damaged tissue when stimulated. In this way, we observed in RAG-KO mice a second set of lymphocytes that arrived in the injured paw near two weeks after nerve crushing, possibly enhancing local inflammatory response and causing hyperalgesia and transient loss of movements. One interesting finding was that at 21 days after crushing, when regeneration is advanced and the anti-inflammatory response is predominant (Mietto et al., 2015), a second peak of hyperalgesia was observed, without increased lymphocyte influx in the paw. Of note, during the same period the nerve is under an intense process of tissue remodeling, and the newly regenerated sensory fibers may have a low pain threshold (Zhang et al., 1997), responsible for the hyperalgesia. However, many surplus and non-functional axonal sprouts are still present in the regenerating nerve, and should be eliminated. Thus, a second wave of degeneration occurs in the nerve (Liuzzi and Tedeschi, 1991; Madison et al., 1996) and, apparently, the adaptive immunity acts in the tissue remodeling process, as evidenced by the third peak of lymphocyte infiltration in the paw between days 21 and 35 after injury. Finally, transference of lymphocytes 14 days after nerve crushing resulted in no further overall improvement in both mouse strains, possibly due to the completion of Wallerian degeneration at such moment, accompanied by a decreased inflammatory response.

Overall, the present data indicate that the modulation of the immune response is a key strategy for achieving better motor recovery following peripheral nerve injury. In line with this, suppression of immune activation by regulatory T cells is neuroprotective in a mouse model of Parkinson’s disease (Reynolds et al., 2007) and acute experimental stroke (Liesz et al., 2009). Shifting the pro-inflammatory Th1 response to the anti-inflammatory Th2 can also promote neuroprotection. In this way, the administration of the Th2 inducer glatiramer acetate increased synapse stability during EAE (Scorisa et al., 2011) while simvastatin, another Th2 inducer, was shown to improve functional recovery of the crushed sciatic nerve in rats, as well as to decrease mononuclear cell infiltrate and edema areas in the damaged tissue (Xavier et al., 2012). Herein, mice with enhanced innate immune capacity presented improved axonal growth and no transient impairments in the motor recovery, which was further ameliorated by the transference of activated lymphocytes in the early stage of the Wallerian degeneration. Such findings may in turn provide the basis for a more effective immune response based therapy following peripheral injury in patients.

## Author Contributions

AB, JS and RT conceived and designed experiments, acquired, analyzed and interpreted data and wrote the manuscript. SN, BM, EF and IB contributed to acquiring, analyzing and interpreting data. CS and LV contributed to data comprehension and wrote the manuscript. AR conceived and designed experiments, interpreted data and wrote the manuscript. All authors read and approved the final manuscript.

## Conflict of Interest Statement

The authors declare that the research was conducted in the absence of any commercial or financial relationships that could be construed as a potential conflict of interest.
