# Supplementary material for: Enhanced Immune Response in Immunodeficient Mice Improves Peripheral Nerve Regeneration Following Axotomy
Source: Front Cell Neurosci. 2016 Jun 14;10:151. doi: 10.3389/fncel.2016.00151 (PMC4905955; doi:10.3389/fncel.2016.00151)
Supplement: Supplementary file 1 [file Table_1.DOCX]

**Table S1. Neurofilament quantification (lesioned/unlesioned)**

| Time  (wal) | WT | | | RAG-KO | | |
| --- | --- | --- | --- | --- | --- | --- |
|  | Mean | SE | N | Mean | SE | N |
| 0 | 1.067 | 0.090 | 6 | 1.000 | 0.036 | 6 |
| 2 | 0.575 | 0.094 | 6 | 0.866 | 0.133 | 6 |
| 4 | 1.355 | 0.172 | 6 | 0.769 | 0.065 | 6 |
| 8 | 2.064 | 0.223 | 6 | 0.915 | 0.027 | 6 |

wal, weeks after lesion
