# Supplementary material for: Enhanced Immune Response in Immunodeficient Mice Improves Peripheral Nerve Regeneration Following Axotomy
Source: Front Cell Neurosci. 2016 Jun 14;10:151. doi: 10.3389/fncel.2016.00151 (PMC4905955; doi:10.3389/fncel.2016.00151)
Supplement: Supplementary file 2 [file Table_2.DOCX]

**Table S2. GAP43 quantification (integrated density of pixels)**

| Time  (wal) | WT | | | RAG-KO | | |
| --- | --- | --- | --- | --- | --- | --- |
|  | Mean | SE | N | Mean | SE | N |
| 0 | 43996 | 10023 | 6 | 5.591 x 10^6^ | 1.764 x 10^6^ | 6 |
| 2 | 222075 | 114081 | 6 | 2.674 x 10^7^ | 5.786 x 10^6^ | 6 |
| 4 | 759147 | 447726 | 6 | 2.174 x 10^6^ | 1.301 x 10^6^ | 6 |
| 8 | 2.183 x 10^6^ | 1.195 x 10^6^ | 6 | 43976 | 21237 | 6 |

wal, weeks after lesion
