# Supplementary material for: Enhanced Immune Response in Immunodeficient Mice Improves Peripheral Nerve Regeneration Following Axotomy
Source: Front Cell Neurosci. 2016 Jun 14;10:151. doi: 10.3389/fncel.2016.00151 (PMC4905955; doi:10.3389/fncel.2016.00151)
Supplement: Supplementary file 3 [file Table_3.DOCX]

**Table S3. Iba1 quantification (integrated density of pixels)**

| Time  (wal) | WT | | | RAG-KO | | |
| --- | --- | --- | --- | --- | --- | --- |
|  | Mean | SE | N | Mean | SE | N |
| 0 | 39807 | 20565 | 6 | 2.037 x 10^6^ | 192258 | 6 |
| 2 | 2.204 x 10^7^ | 1.022 x 10^6^ | 6 | 2.923 x 10^7^ | 1.604 x 10^6^ | 6 |
| 4 | 5.669 x 10^6^ | 1.722 x 10^6^ | 6 | 8.560 x 10^6^ | 3.046 x 10^6^ | 6 |
| 8 | 2.441 x 10^6^ | 191576 | 6 | 152176 | 80608 | 6 |

wal, weeks after lesion
