# Supplementary material for: Enhanced Immune Response in Immunodeficient Mice Improves Peripheral Nerve Regeneration Following Axotomy
Source: Front Cell Neurosci. 2016 Jun 14;10:151. doi: 10.3389/fncel.2016.00151 (PMC4905955; doi:10.3389/fncel.2016.00151)
Supplement: Supplementary file 4 [file Table_4.DOCX]

**Table S4. Thigh lymphocyte labeling area (mm²)**

| Time  (dal) | WT | | | RAG-KO | | |
| --- | --- | --- | --- | --- | --- | --- |
|  | Mean | SE | N | Mean | SE | N |
| 3 | 119.00 | 19.35 | 3 | 115.90 | 29.99 | 5 |
| 4 | 75.09 | 7.69 | 3 | 93.22 | 22.48 | 5 |
| 7 | 56.33 | 7.75 | 3 | 7.75 | 4.84 | 5 |

dal, days after lesion
