# Supplementary material for: Enhanced Immune Response in Immunodeficient Mice Improves Peripheral Nerve Regeneration Following Axotomy
Source: Front Cell Neurosci. 2016 Jun 14;10:151. doi: 10.3389/fncel.2016.00151 (PMC4905955; doi:10.3389/fncel.2016.00151)
Supplement: Supplementary file 5 [file Table_5.DOCX]

**Table S5. Paw lymphocyte labeling area (mm²)**

| Time  (dal) | WT | | | RAG-KO | | |
| --- | --- | --- | --- | --- | --- | --- |
|  | Mean | SE | N | Mean | SE | N |
| 3 | 26.92 | 8.33 | 3 | 14.34 | 4.37 | 5 |
| 4 | 26.96 | 8.13 | 3 | 21.18 | 6.25 | 5 |
| 7 | 26.55 | 7.95 | 3 | 22.45 | 4.84 | 5 |
| 10 | 16.43 | 6.47 | 3 | 9.70 | 3.28 | 5 |
| 13 | 25.56 | 4.58 | 3 | 17.30 | 6.91 | 5 |
| 14 | 20.62 | 1.02 | 3 | 14.19 | 2.58 | 5 |
| 18 | 22.53 | 3.51 | 3 | 10.13 | 2.98 | 5 |
| 21 | 9.87 | 2.08 | 3 | 6.60 | 2.61 | 5 |
| 24 | 19.67 | 6.10 | 3 | 8.24 | 2.82 | 5 |
| 28 | 17.01 | 3.28 | 3 | 10.05 | 4.45 | 5 |
| 31 | 12.98 | 1.48 | 3 | 2.71 | 1.00 | 5 |
| 35 | 10.83 | 3.20 | 3 | 3.78 | 1.27 | 5 |

dal, days after lesion
